# The health facility as a risk factor for multidrug-resistant gram-negative bacteria in critically ill patients with COVID-19

**DOI:** 10.1016/j.clinsp.2022.100130

**Published:** 2022-10-17

**Authors:** Viviane de Macedo, Gabriela de Souza dos Santos, Rodolff Nunes da Silva, Caio Nogara de Menezes Couto, Camila Bastos, Eloize Viecelli, Marina do Nascimento Mateus, Maria Esther Graf, Raquel Bernardelli Gonçalves, Márcia Aparecida da Silva, Patricia Dal Bem Bernardini, Roberta Serra Pereira Grando, Viviane Pavanelo Boaventura, Helki Simone Rodrigues Pereira, Anna S. Levin

**Affiliations:** a Hospital Infection Control and Epidemiology Center, Santa Casa de Curitiba, Curitiba, PR, Brazil; b Faculdade de Medicina, Universidade Positivo, Curitiba, PR, Brazil; c Department of Infectious Diseases, Faculdade de Medicina, Universidade de São Paulo, São Paulo, SP, Brazil; d Hospital Infection Control and Epidemiology Center, Rehabilitation Hospital, Curitiba, PR, Brazil; e Infection Control Program, Hospital de Clínicas, Curitiba, PR, Brazil; f Faculdade de Medicina, Pontifícia Universidade Católica do Paraná, Curitiba, PR, Brazil; g Hospital Infection Control and Epidemiology Center, Hospital do Trabalhador, Curitiba, PR, Brazil

**Keywords:** COVID-19, Antimicrobial resistance, Gram-negative bacteria, Intensive Care Unit, Health Facility

## Abstract

**Background:**

The relationship between Multidrug Resistant-Gram Negative Bacteria (MDR-GNB) infection and colonization in critically ill COVID-19 patients has been observed, however, it is still poorly understood. This study evaluated the risk factors for acquiring MDR-GNB in patients with severe COVID-19 in Intensive Care Units (ICU).

**Methods:**

This is a nested case-control study in a cohort of 400 adult patients (≥ 18 years old) with COVID-19, hospitalized in the ICU of 4 hospitals in the city of Curitiba, Brazil. Cases were critical COVID-19 patients with one or more MDR GNB from any surveillance and/or clinical cultures were taken during their ICU stay. Controls were patients from the same units with negative cultures for MDR-GNB. Bivariate and multivariate analyses were done.

**Results:**

Sixty-seven cases and 143 controls were included. Independent risk factors for MDR bacteria were: male gender (OR = 2.6; 95% CI 1.28‒5.33; p = 0.008); the hospital of admission (OR = 3.24; 95% CI 1.39‒7.57; p = 0.006); mechanical ventilation (OR = 25.7; 95% CI 7.26‒91; p < 0.0001); and desaturation on admission (OR = 2.6; 95% CI 1.27‒5.74; p = 0.009).

**Conclusions:**

Male gender, desaturation, mechanical ventilation, and the hospital of admission were the independent factors associated with MDR-GNB in patients in the ICU with COVID-19. The only modifiable factor was the hospital of admission, where a newly opened hospital posed a higher risk. Therefore, coordinated actions toward a better quality of care for critically ill COVID-19 patients are essential.

## Highlights

• Critically ill patients with COVID-19 can have secondary infections.
• Some of them are caused by Multidrug-Resistant (MDR) Gram-Negative Bacteria (GNB).
• To know the risk factors related to MDR-GNB is essential to analyze if it is possible to prevent them.
• Hospital of admission for patients with COVID-19 posed as high risk for MDR-GNB.
• This is a modifiable risk factor.

## Introduction

Bacterial Antimicrobial Resistance (AMR) is one of the main threats to global health, limiting therapeutic options for treating infectious diseases. This leads to longer hospital stays, higher medical costs, and increased mortality.^1^ Before the COVID-19 pandemic, there were an estimated 4.95 million deaths associated with bacterial AMR in 2019, including 1.27 million deaths attributable to bacterial AMR in the world.^2^

Mainly during the first and second waves of the COVID-19 pandemic, there was excessive use of antimicrobials due to the severity of the patients and the lack of therapeutic options. Overuse of antibiotics has been related to antimicrobial resistance.^3^ Furthermore, hospitals were overcrowded and with overwhelming shortages in essential resources: healthcare workers, Personal Protective Equipment (PPE), ventilators, hospital beds, and infection control programs.^4^ Overcrowding has been associated with lower quality of care and a higher prevalence of infectious diseases among patients.^5^

Studies have shown that mainly critically ill patients with COVID-19 have secondary infections,^6^, ^7^, ^8^, ^9^, ^10^ a proportion of them caused by Multidrug-Resistant (MDR) Gram-Negative Bacteria (GNB). The incidence of MDR-GNB infections in critically ill COVID-19 patients in different regions of the world ranges between 3.4% to 46%.^6^, ^7^, ^8^, ^9^, ^10^, ^11^, ^12^, ^13^, ^14^, ^15^ COVID-19 patients admitted to the ICU also seem to be more susceptible to colonization by MDR.^16^

Although MDR-GNB infections and colonizations have been linked to prolonged length of stay in the ICU, longer invasive mechanical ventilation, and steroid therapy,^7^,^16^ this relation is not fully understood and has been still little explored. Therefore, the aim of this study was to identify the risk factors for the isolation of MDR-GNB in critically ill COVID-19 patients.

## Methods

This is a nested case-control study in a cohort of 400 adult patients (≥ 18 years old) with COVID-19 hospitalized in the Intensive Care Units (ICU) of 4 hospitals in the city of Curitiba, Brazil:^8^ 1- Santa Casa (a philanthropic hospital with 249 beds, with 48 ICU beds, which is a referral hospital for chest pain, and renal and heart transplantation); 2- Trabalhador Hospital (a state institution with 222 beds, including 40 ICU beds, that is a trauma referral hospital); 3- Rehabilitation Hospital (a state institution with 82 beds, including 62 ICU beds, which was adapted for COVID-19 patients only); and 4- Institute of Medicine (a private hospital, which was reopened by the city as a field hospital for COVID-19 patients, with 100 beds, including 60 ICU beds). The patients were admitted between 1^st^ March and 31^st^ December 2020.

Cases were defined as severe COVID-19 patients with one or more MDR GNB from any surveillance and/or clinical cultures taken during their ICU stay.

Controls were severe COVID-19 patients in which no MDR GNB and/or MDR gram-positive bacteria were isolated (surveillance and/or clinical cultures) during their ICU stay. Patients who did not undergo surveillance cultures were excluded from the control group.

COVID-19 was defined as an infection confirmed with a positive Real-Time reverse transcriptase Polymerase Chain Reaction (RT-PCR) for SARS-CoV-2 from a nasopharyngeal swab, associated with suggestive signs, symptoms, and/or compatible radiological findings.

Patients with severe disease were defined as patients with Oxygen Saturation (SpO_2_) ≤94% in ambient air; or requiring supplemental oxygen; requiring mechanical ventilation; or requiring extracorporeal membrane oxygenation.^8^

The WHO Clinical Progression Scale^17^ was used to measure the COVID-19 severity of each patient on admission to the ICU: 0 (not infected); 1‒3 (ambulatory mild disease): 1- Asymptomatic; 2- Symptomatic, independent; 3- Symptomatic, assistance needed; 4‒5 (hospitalized with moderate disease): 4- No oxygen needed; 5- Oxygen by mask or nasal prongs; 6‒9 (hospitalized: severe disease) 6- Oxygen by non-invasive ventilation or high flow; 7- Intubation and mechanical ventilation, pO_2_/FiO_2_ ≥150 or SpO_2_/FiO_2_ ≥200; 8- Mechanical ventilation pO_2_/FiO_2_ < 150 or SpO_2_/FiO_2_ < 200, or vasopressors; 9- Mechanical ventilation pO_2_/FiO_2_ < 150 and vasopressors, dialysis, or ECMO; and 10 (dead).

The following GNBs were considered to be Multidrug Resistant (MDR):

Enterobacteriales resistant from first to third/fourth generation cephalosporins;
Carbapenem-Resistant Enterobacteriales (CRE);
Carbapenem-Resistant *Acinetobacter* spp. (CRA);
Carbapenem-Resistant *Pseudomonas aeruginosa* (CRP).

For bacterial identification, MICROSCAN WalkAway® (Beckman Coulter, Inc., Brea, CA, USA) automated system was used. Sensitivity to antimicrobial agents was also determined by MICROSCAN WalkAway® system according to the Brazilian Committee on Antimicrobial Susceptibility (BRCast) criteria. The phenotypic confirmation of ESBL production was done using the double-disk synergy test with cefotaxime, ceftazidime, and cefepime with and without clavulanic acid. Combined disk diffusion using phenylboronic acid as an inhibitor with cefoxitin was used for phenotypic confirmation of AmpC phenotype. Carbapenemase production was demonstrated by the modified Hodge test and by the Modified Carbapenem Inactivation Method (mCIM) and EDTA-mCIM (eCIM).

Broad-spectrum antibiotics were defined as any agent with activity against gram-positive bacteria such as Methicillin-Susceptible *Staphylococcus Aureus* (MSSA) and gram-negative bacteria such as Enterobacteriales and *Pseudomonas*. These included piperacillin-tazobactam, meropenem, cefepime, ceftazidime, amikacin, gentamicin, and fluoroquinolones.^18^

Data were extracted from electronic medical records using a data collection form. Demographic characteristics included age, sex, weight, and Body Mass Index (BMI). Clinical information included signs, symptoms, comorbidities, and treatment measures (antimicrobial therapy; steroid therapy; respiratory support; use of kidney replacement therapy; and length of use of invasive devices such as an orotracheal tube, central venous catheter, and indwelling urinary catheter). Laboratory assessment consisted of laboratory tests on admission to the ICU and at the moment of diagnosis of a secondary infection: C-reactive protein, total leukocyte count, number of lymphocytes, creatinine, glucose, D-dimer, troponin, and the ratio of Arterial Oxygen Partial pressure (PaO_2_) to Fraction of Inspired Oxygen (FiO_2_). Lung radiologic alterations were defined based on the medical report of the chest radiograph or computed tomography. Duration of the disease from the onset of symptoms, and length of stay in the hospital and in the ICU was also documented.

This study was approved by the research ethics committees of the study hospitals (Protocol n° 4.361.502, CAAE: 38239820.8.0000.5225).

### Data analysis

The authors compared all critical COVID-19 patients with one or more MDR GNB from any surveillance and/or clinical cultures taken during their ICU admission (cases), with the controls.

The possible association of demographic and clinical variables with each dependent variable was initially tested in a bivariate analysis calculating the odds ratios and 95% Confidence Interval for each variable. Variables potentially associated with the development of each dependent variable in bivariable analysis (p < 0.20) were included in a multivariable logistic regression model in order to determine the adjusted odds ratios. Mechanical ventilation was chosen to represent invasive devices in multivariable analysis.

The variables C-reactive Protein (CRP), PaO_2_/FiO_2_ ratio, absolute lymphocyte count, and absolute leukocyte count were transformed from continuous variables to categorical variables. For CRP, the cutoff point used was the value of 108 mg/L, based on the study ^19^ which observed an increase in mortality among patients with values above this cut-off. For PaO_2_/FiO_2_ four categories of severity of acute respiratory syndrome were used, according to Villar et al.^20^ namely: >300 mmHg; 200‒299 mmHg; 100‒199 mmHg, and < 100 mmHg. For absolute lymphocyte count, the cutoff point used was the value of < 1.0 × 10^3^ cells/µL, based on Wagner et al.,^21^ which observed that values below this cut-off were related to disease severity and clinical outcomes in COVID-19. For absolute leukocyte count, leukocytosis was defined as a leukocyte count >11.0 × 10^3^ cells/µL.^22^

Statistical analyses were performed using EPI Info 7 Software (version 7.2.4, Centers for Disease Control and Prevention, Atlanta, EUA); p-values < 0.05 were considered to be statistically significant.

## Results

From a cohort of 400 adult patients, 67 cases (15 with surveillance cultures positive for MDR GNB; 34 with clinical cultures positive for MDR-GNB; and 18 positives for both surveillance and clinical cultures) and 143 controls were included.

Among the demographic characteristics, clinical information, treatment measures, and complementary exams, the factors associated with MDR-GNB in the bivariate analysis were: gender; the hospital of admission; WHO Clinical Progression Scale; low leukocyte count; low lymphocyte count; high C-reactive protein; low PaO_2_/FiO_2_ ratio on ICU admission; pronation; and use of devices in the ICU (Table 1).

**Table 1:** Factors associated with acquiring multidrug-resistant gram-negative bacteria in patients with severe COVID-19 admitted to intensive care units of 4 hospitals (Curitiba, Brazil. March ‒ December 2020).

Characteristics	Cases (n = 67)	Controls (n = 143)	Bivariate analysis Odds Ratio (95% CI)	p*	Multivariate analysis, Odds Ratio (95% CI)	p*
Age, years mean (SD)	62.6 (13.6)	64.6 (14.6)		0.34		
Gender, n (%)						
Female	23 (34)	71 (50)	Reference			
Male	44 (66)	72 (50)	1.88 (1.03‒3.44)	0.03	2.61 (1.28‒5.33)	0.008
Hospital of admission						
Santa Casa	12 (18)	51 (36)	Reference			
Trabalhador Hospital	13 (19)	35 (24)	1.50 (0.64‒3.86)	0.31	3.24 (1.39‒7.57)	0.0064
Institute of Medicine	20 (30)	20 (14)	4.20 (1.75‒10.2)	0.0013		
Rehabilitation Hospital	22 (33)	37 (26)	2.50 (1.11‒5.74)	0.02		
Comorbidities, n (%)						
Hypertension	47 (70)	87 (61)	1.50 (0.81‒2.81)	0.19		
Coronary heart disease	20 (30)	37 (26)	1.20 (0.64‒2.31)	0.54		
Diabetes	20 (30)	49 (34)	0.81 (0.43‒1.52)	0.52		
Chronic kidney disease	5 (7)	11 (8)	0.96 (0.32‒2.90)	0.95		
Chronic obstructive lung disease	8 (12)	13 (9)	1.30 (0.53‒3.44)	0.52		
Cancer	2 (3)	4 (3)	1.06 (0.19‒5.90)	0.93		
Body Mass Index, n (%)						
18‒24.9	6 (14)	16 (19)				
25‒29.9	15 (34)	33 (40)	1.17 (0.38‒3.50)	0.77		
≥30	23 (52)	33 (40)	1.85 (0.63‒5.40)	0.26		
Signs and symptoms on hospital admission, n (%)						
Cough	41 (61)	99 (69)	0.70 (0.38‒1.28)	0.25		
Dyspnoea	58 (86)	115 (80)	1.56 (0.69‒3.54)	0.27		
Fever ≥ 37°C	35 (52)	73 (51)	1.04 (0.58‒1.87)	0.87		
Desaturation (SpO_2_ < 92%)	48 (72)	87 (61)	1.62 (0.86‒3.04)	0.12	2.70 (1.27‒574)	0.0009
Fatigue	15 (22)	36 (25)	0.85 (0.43‒1.70)	0.66		
Diarrhea	2 (3)	12 (8)	0.33 (0.07‒1.54)	0.16		
Nausea or vomiting	3 (4)	13 (9)	0.47 (0.13‒1.71)	0.25		
Headache	3 (4)	15 (10)	0.43 (0.11‒1.44)	0.16		
Anosmia	8 (12)	18 (12)	0.94 (0.38‒2.28)	0.89		
Dysgeusia	5 (7)	15 (10)	0.68 (0.23‒1.97)	0.49		
Sore throat	1 (1)	7 (5)	0.29 (0.03‒2.44)	0.25		
Treatment and outcomes						
Pronation, n (%)	40 (65)	106 (38)	0.51 (0.27‒0.95)	0.035		
Renal replacement therapy, n (%)	21 (28)	43 (16)	1.06 (0.56‒1.98)	0.85		
MV, n (%)	64 (95)	74 (52)	9.50 (3.16‒25)	<0.0001	25.7 (7.26‒91)	<0.0001
Mean days MV (SD)	11 (7)	8(5)		0.0005		
CVC, n (%)	64 (95)	74 (52)	9.50 (3.16‒25)	<0.0001		
Mean days of CVC (SD)	10 (5)	7 (5)		0.0001		
IUC, n (%)	64 (95)	82 (57)	15.80 (4.7‒52)	<0.0001		
Mean days of IUC (SD)	10 (5)	9 (5)		0.17		
Use of steroids, n (%)	50 (78)	103 (72)	1.14 (0.59‒2.21)	0.69		
Use of anticoagulants, n (%)	67 (100)	138 (96)	5.36 (0.29‒98)	0.25		
Therapeutic dose	3 (5)	16 (11)	0.37 (0.10‒1.32)	0.12		
Prophylactic dose	64 (95)	122 (85)	3.67 (1.05‒12.7)	0.04		
Use of antibiotics, n (%)	63 (94)	139 (97)	0.45 (0.10‒1.87)	0.27		
Broad-spectrum antibiotics, n (%)	22 (33)	32 (22)	1.69(0.89‒3.20)	0.10		
Ceftriaxone	55 (87)	124 (89)	0.7 (0.31‒1.50)	0.38		
Azithromycin	63 (94)	120 (86)	3.0 (1.00‒9.10)	0.05		
Cefepime	11 (17)	20 (14)	1.2 (0.54‒2.60)	0.64		
Piperacillin-tazobactam	10 (16)	8 (6)	2.9 (1.11‒7.80)	0.02		
Amikacin	7 (11)	9 (5)	1.7 (0.61‒4.80)	0.29		
Meropenem	2 (3)	2 (1)	2.1 (0.29‒15.7)	0.44		
Levofloxacin	4 (6)	1 (0.38)	9.01 (0.98‒82)	0.05		
Vancomycin	8 (13)	7 (5)	2.4 (0.84‒7.04)	0.09		
WHO scale on admission ‒ Mean (SD)	6.0 (1.25)	5.3 (1.10)		0.0001		
Days between onset of symptoms and ICU admission – Mean (SD)	6.9 (4.90)	7.2 (5.10)		0.88		
Days between hospital and ICU admission ‒ Mean (SD)	1.1 (2.70)	1.1 (2.40)		0.96		
Days between ICU admission and death ‒ Mean (SD)	18.5 (12.5)	8.8 (5.4)		<0.0001		
Death, n (%)	48 (72)	53 (37)	4.3 (2.28‒8.00)	<0.0001		
Laboratory test results on admission to the ICU ‒ Mean (SD)						
Leukocyte count (cells/mm^3^)	10921 (4113)	12705 (6341)		0.03		
Lymphocyte count (cells/mm^3^)	957 (401)	1559 (730)		<0.0001		
C-reactive protein (mg/L)	170 (73)	106 (83)		<0.0001		
Creatinine (mg/dL)	1.49 (0.85)	1.63 (1.1)		0.35		
Blood glucose (mg/dL)	181 (70.4)	186 (89)		0.68		
PaO_2_/FIO_2_ ratio	152 (75)	232 (113)		<0.0001		
Thoracic CT alterations on admission to the ICU, n (%)						
Ground glass opacities	45 (92)	116 (92)	0.96 (0.28‒3.25)	0.96		
Consolidation	12 (24)	20 (16)	1.71 (0.76‒3.85)	0.18		
Atelectasis	8 (16)	26 (21)	0.75 (0.31‒1.79)	0.51		
Interlobular thickening	13 (26)	35 (28)	1.20 (0.56‒2.59)	0.62		
Pleural effusion	7 (14)	22 (17)	0.78(0.31‒1.98)	0.61		

CI, Confidence Interval; CVC, Central Venous Catheter; ICU, Intensive Care Unit; IUC, Indwelling Urinary Catheter; MV, Mechanical Ventilation; OR, Odds Ratio; SD, Standard Deviation; SpO_2_, Oxygen Saturation; WHO, World Health Organization; CT, Computed Tomography.

In the multivariate analysis, the variables that significantly increased the risk of GNB-MDR were: gender, the hospital of admission; the use of mechanical ventilation; and desaturation on hospital admission. Male gender increased the risk 2.5 fold (OR = 2.6; 95% CI 1.28‒5.33; p = 0.008), while the hospital of admission increased the risk by 3 fold (OR = 3.24; 95% CI 1.39‒7.57; p = 0.006). Mechanical ventilation increased the risk of MDR by 26 fold (OR = 25.7; 95% CI 7.26‒91; p < 0.0001) while for desaturation the risk was 2.6 fold (OR = 2.6; 95% CI 1.27‒5.74; p = 0.009).

In surveillance cultures, CR-*Acinetobacter baumannii* was the most frequent bacteria (22 cases – 65%) followed by CR-Enterobacterales (10 cases – 29%) and CR-*Pseudomonas* (2 cases – 6%). In clinical cultures, there was also a predominance of CR-*Acinetobacter baumannii.* In respiratory samples, 15 were CRA; 11 were Enterobacteriales resistant to 3^rd^ and 4^th^ generation cephalosporins; 5 were CRE, and 2 were CRP. In blood cultures, 6 were CRA; 5 were CRE; 3 were Enterobacteriales resistant to 3^rd^ and 4^th^ generation cephalosporins, and 2 were CRP. Finally, in urine cultures, 2 of each were CRA; CRE; and Enterobacteriales resistant to 3^rd^ and 4^th^ generation cephalosporins; and 1 was CRP.

38 cases had healthcare-associated infections caused by MDR-GNB: 28 were lower tract respiratory (22 Ventilator-Associated Pneumonia [VAP], 2 non-ventilator associated pneumonia, and 4 tracheobronchitis); 7 central venous Catheter-related Bloodstream Infections [CLABSI], and 3 Catheter-Associated Urinary Tract Infection (CAUTI)].

Death was higher among the cases (72%) than controls (37%) (OR = 4.3; 95% CI 2.28‒8.0; p < 0.0001).

## Discussion

In this nested case-control study in a cohort of 400 adult patients with COVID-19 admitted to the ICU of 4 hospitals, the authors found that the independent factors associated with MDR-GNB were the hospital of admission, the use of mechanical ventilation, desaturation, and male gender.

Among these four factors, the hospital of admission is the only modifiable variable. This highlights the fact that Infection Prevention and Control (IPC) measures play an essential role in health facilities, making safer places to care for COVID-19 patients, and avoiding colonization, cross-transmission, and infection by MDR bacteria. Even more so because in severe cases of COVID-19 bacterial infection occurs in approximately 13%, much lower than in influenza, which is > 34%.^23^ This suggests that differently from influenza COVID-19 does not lead to a special propensity towards secondary bacterial infections. Taken together, the present findings strengthen the need for health services to implement and to adhere to IPC measures.

The characteristics of the work environment affect the quality of care both directly and indirectly. In the present study, the hospital with the highest risk for MDR-GNB had been closed and was reopened as a field hospital for COVID-19 patients. The structure of the hospital, as well as the ICU, was adapted to receive COVID-19 patients. In addition, there was a mixture of experienced and inexperienced healthcare workers due to a scarcity of specialized personnel. The processes of work were being organized while the patients were being hospitalized, as well as the training of medical and multidisciplinary teams on infection prevention and control: hand hygiene, the use of personal protective equipment, equipment disinfection, and environmental cleaning. Furthermore, the overcrowding of patients may also have affected the implementation of isolation measures for patients colonized with MDR, leading to potential outbreaks.^16^ All these factors made the ideal environment for the increase of MDR bacteria.^15^,^16^

In fact, in the present study *Acinetobacter baumannii* was the most frequent MDR-GNB. This bacterium has the capacity to survive for prolonged periods on inanimate objects and even in hand sanitizers.^24^ Longer ICU stays, and frequent and prolonged use of invasive devices (mechanical ventilation, central venous catheter) favor colonization of patients by MDR bacteria. Furthermore, prolonged use of gloves without adequate hand hygiene plus the use of the same gown, discarded only after an entire work shift ^25^ may favor environmental colonization, especially by *Acinetobacter*.^7^,^8^,^10^,^12^,^14^ Many hospitals in different parts of the world also had difficulty in effectively applying infection control strategies. For example, in Parana, the southern Brazilian state in which this study was carried out, Carbapenem-Resistant *A. baumannii* (CRAB) increased from 7.9% in 2019 to 12.4% in 2020. The incidence of CRAB per 1000 patient days increased significantly after April 2020 and correlated strongly with the incidence of COVID-19.^26^

Many studies have shown the correlation between MDR and mechanical ventilation and/or the use of invasive devices in patients with COVID-19.^27^,^28^ Baiou A. et al.^15^ observed the only risk factor independently associated with MDR infections was mechanical ventilation. A pre-pandemic meta-analysis^29^,^30^ also showed that ventilation (within the previous 6 months)^30^ was significantly associated with the increase of MDR bacteria. Invasive procedures affecting the respiratory tract and the prolonged use of an endotracheal tube can damage the respiratory mucosa. Furthermore, the prolonged use of mechanical ventilation may make sputum dry and sticky which can also increase the risk of lung infection and injury. Interference with normal respiratory barrier and physiological functions increases the likelihood of MDR‐GNB infection and increases the incidence of ventilator‐associated pneumonia.^31^

In the present study, patients with desaturation on hospital admission had a higher risk for MDR bacteria. Patients with severe COVID-19 have a dysfunctional immune response, which triggers a cytokine storm that mediates widespread lung inflammation that in itself mediates damage to the lung through excessive secretion of proteases and reactive oxygen species, in addition to the direct damage caused by the virus. Together, these result in diffuse alveolar damage, including desquamation of alveolar cells, hyaline membrane formation, and pulmonary edema. This limits the efficiency of gas exchange in the lung, causing difficulty in breathing and low blood oxygen levels. The lung also becomes more vulnerable to secondary infections, among them MDR-GNB, mainly when adequate environmental cleaning and hand hygiene are lacking.^32^

Finally, it is not clear why MDR-GNB is more prevalent in men.^31^,^33^,^34^ Some explanations may be physical function, lifestyle (higher smoking rate, for example), weaker innate and adaptive immune system, and male sex hormones. Testosterone has an immunosuppressive behavior, while females have more immunity-related genes on the X chromosome, and estrogen may help them combat and prevent different diseases.^35^,^36^ Finally, men have more comorbidities when compared to women, and have been shown to have a poor medical follow-up.^37^ All these factors may lead to prolonged hospitalization, which may favor colonization by MDR bacteria.

The strength of this study is its multicenter design, although only hospitals in one city were included, and the relatively large number of cases and controls. However, many patients could not be included as controls due to the absence of surveillance cultures to ascertain that they were not cases

## Conclusions

In conclusion, in the present study, male gender, desaturation, mechanical ventilation, and the hospital of admission were the independent factors associated with MDR-GNB in patients in ICU with COVID-19. The only modifiable factor was the hospital of admission, as the newly opened hospital posed a higher risk. Therefore, efforts are needed to improve the quality of care to patients with severe COVID-19 in order to avoid or reduce colonization and infection by MDR-GNB bacteria. These findings also suggest that better public health preparedness for emergencies in Brazil is necessary. Good quality of health care should be the focus of robust actions in order to mitigate the transmission of MDR-GNB bacteria.

## Conflicts of interest

The authors declare no conflicts of interest.

## References

[1] WHO. Antimicrobial resistance. 2022. https://www.who.int/newsroom/fact-sheets/detail/antimicrobial-resistance (accessed Jun 16, 2022).

[2] Antimicrobial Resistance Collaborators (2022). Global burden of bacterial antimicrobial resistance in 2019: a systematic analysis. Lancet.

[3] Rawson TM, Moore LSP, Zhu N, Ranganathan N, Skolimowska K, Gilchrist M (2020). Bacterial and fungal coinfection in individuals with coronavirus: a rapid review to support COVID-19. Antimicrobial Prescribing. Clin Infect Dis.

[4] Badalov E, Blackler L, Scharf AE, Matsoukas K, Chawla S, Voigt LP (2022). COVID-19 double jeopardy: the overwhelming impact of the social determinants of health. Int J Equity Health.

[5] Virtanen M, Terho K, Oksanen T, Kurvinen T, Pentti J, Routamaa M (2011). Patients with infectious diseases, overcrowding, and health in hospital staff. Arch Intern Med.

[6] Bongiovanni M, Barilaro G, Zanini U, Giuliani G. (2022). Impact of the COVID-19 pandemic on multidrug-resistant hospital-acquired bacterial infections. J Hosp Infect.

[7] Li J, Wang J, Yang Y, Cai P, Cao J, Cai X (2020). Etiology and antimicrobial resistance of secondary bacterial infections in patients hospitalized with COVID-19 in Wuhan, China: A retrospective analysis. Antimicrob Resist Infect Control.

[8] de Macedo V, Santos GS, Silva RN, Couto CNM, Bastos C, Viecelli E (2022). Healthcare-associated infections: a threat to the survival of patients with COVID-19 in intensive care units. J Hosp Infect.

[9] Pasero D, Cossu AP, Terragni P. (2021). Multi-drug resistance bacterial infections in critically ill patients admitted with COVID-19. Microorganisms.

[10] Costa RLD, Lamas CDC, Simvoulidis LFN, Espanha CA, Moreira LPM, Bonancim RAB (2022). Secondary infections in a cohort of patients with COVID-19 admitted to an intensive care unit: impact of gram-negative bacterial resistance. Rev Inst Med Trop Sao Paulo.

[11] Grasselli G, Greco M, Zanella A, Albano G, Antonelli M, Bellani G (2020). Risk factors associated with mortality among patients with COVID-19 in intensive care units in Lombardy, Italy. JAMA Intern Med.

[12] Cultrera R, Barozzi A, Libanore M, Marangoni E, Pora R, Quarta B. (2021). Co-infections in critically ill patients with or without COVID-19: a comparison of clinical microbial culture findings. Int J Environ Res Public Health.

[13] Grasselli G, Scaravilli V, Mangioni D, Scudeller L, Alagna L, Bartoletti M (2021). Hospital-acquired infections in critically ill patients with COVID-19. Chest.

[14] Ripa M, Galli L, Poli A, Oltolini C, Spagnuolo V, Mastrangelo A (2021). Secondary infections in patients hospitalized with COVID-19: incidence and predictive factors. Clin Microbiol Infect.

[15] Baiou A, Elbuzidi AA, Bakdach D, Zaqout A, Alarbi KM, Bintaher AA (2021). Clinical characteristics and risk factors for the isolation of multi-drug-resistant Gram-negative bacteria from critically ill patients with COVID-19. J Hosp Infect.

[16] Fernández P, Moreno L, Yagüe G, Andreu E, Jara R, Segovia M. (2021). Colonization by multidrug-resistant microorganisms in ICU patients during the COVID-19 pandemic. Med Intensiva (Engl Ed).

[17] (2020). WHO Working Group on the Clinical Characterisation and Management of COVID-19 infection. A minimal common outcome measure set for COVID-19 clinical research. Lancet Infect Dis.

[18] Hagiya H, Kokado R, Ueda A, Okuno H, Morii D, Hamaguchi S (2019). Association of adverse drug events with broad-spectrum antibiotic use in hospitalized patients: a single-center study. Intern Med.

[19] Smilowitz NR, Kunichoff D, Garshick M, Shah B, Pillinger M, Hochman JS (2021). C-reactive protein and clinical outcomes in patients with COVID-19. Eur Heart J.

[20] Villar J, Pérez-Méndez L, Blanco J, Añón JM, Blanch L, Belda J (2013). Spanish initiative for epidemiology, stratification, and therapies for ARDS (SIESTA) network. A universal definition of ARDS: the PaO_2_/FiO_2_ ratio under a standard ventilatory setting ‒ a prospective, multicenter validation study. Intensive Care Med.

[21] Wagner J, DuPont A, Larson S, Cash B, Farooq A. (2020). Absolute lymphocyte count is a prognostic marker in COVID-19: a retrospective cohort review. Int J Lab Hematol.

[22] Zhao K, Li R, Wu X, Zhao Y, Wang T, Zheng Z (2020). Clinical features in 52 patients with COVID-19 who have increased leukocyte count: a retrospective analysis. Eur J Clin Microbiol Infect Dis.

[23] Chertow DS, Memoli MJ. (2013). Bacterial coinfection in influenza: a grand rounds review. JAMA.

[24] Asif M, Alvi IA, Rehman SU. (2018). Insight into Acinetobacter baumannii: pathogenesis, global resistance, mechanisms of resistance, treatment options, and alternative modalities. Infect Drug Resist.

[25] Polly M, de Almeida BL, Lennon RP, Cortês MF, Costa SF, Guimarães T. (2022). Impact of the COVID-19 pandemic on the incidence of multidrug-resistant bacterial infections in an acute care hospital in Brazil. Am J Infect Control.

[26] de Carvalho Hessel Dias VM, Tuon F, de Jesus Capelo P, Telles JP, Fortaleza CMCB, Pellegrino Baena C. (2022). Trend analysis of carbapenem-resistant Gram-negative bacteria and antimicrobial consumption in the post-COVID-19 era: an extra challenge for healthcare institutions. J Hosp Infect.

[27] Mazzariol A, Benini A, Unali I, Nocini R, Smania M, Bertoncelli A (2021). Dynamics of SARS-CoV2 infection and multi-drug resistant bacteria superinfection in patients with assisted mechanical ventilation. Front Cell Infect Microbiol.

[28] Jamnani AN, Montazeri M, Mirzakhani M, Moosazadeh M, Haghighi M. (2022). Evaluation of bacterial coinfection and antibiotic resistance in patients with COVID-19 under mechanical ventilation. SN Compr Clin Med.

[29] Chen G, Xu K, Sun F, Sun Y, Kong Z, Fang B. (2020). Risk factors of multidrug-resistant bacteria in lower respiratory tract infections: a systematic review and meta-analysis. Can J Infect Dis Med Microbiol.

[30] Zhu WM, Yuan Z, Zhou HY. (2020). Risk factors for carbapenem-resistant Klebsiella pneumoniae infection relative to two types of control patients: a systematic review and meta-analysis. Antimicrob Resist Infect Control.

[31] Ang H, Sun X. (2018). Risk factors for multidrug-resistant Gram-negative bacteria infection in intensive care units: a meta-analysis. Int J Nurs Pract.

[32] Tay MZ, Poh CM, Rénia L, MacAry PA, Ng LFP. (2020). The trinity of COVID-19: immunity, inflammation and intervention. Nat Rev Immunol.

[33] Perez S, Innes GK, Walters MS, Mehr J, Arias J, Greeley R (2020). Increase in hospital-acquired carbapenem-resistant Acinetobacter baumannii infection and colonization in an acute care hospital during a surge in COVID-19 admissions ‒ New Jersey, February-July 2020. MMWR Morb Mortal Wkly Rep.

[34] Karruli A, Boccia F, Gagliardi M, Patauner F, Ursi MP, Sommese P (2021). Multidrug-resistant infections and outcome of critically ill patients with coronavirus disease 2019: a single center experience. Microb Drug Resist.

[35] Jaillon S, Berthenet K, Garlanda C. (2019). Sexual dimorphism in innate immunity. Clin Rev Allergy Immunol.

[36] Chen N, Zhou M, Dong X, Qu J, Gong F, Han Y (2020). Epidemiological and clinical characteristics of 99 cases of 2019 novel coronavirus pneumonia in Wuhan, China: a descriptive study. Lancet.

[37] Ejaz R, Ashraf MT, Qadeer S, Irfan M, Azam A, Butt S (2021). Gender-based incidence, recovery period, and mortality rate of COVID-19 among the population of district Attock, Pakistan. Braz J Biol.

